# Long-read sequencing reveals absence of 5mC in Ogataea parapolymorpha DL-1 genome and introduces telomere-to-telomere assembly

**DOI:** 10.3389/fgene.2025.1574332

**Published:** 2025-05-09

**Authors:** Andrey Eremin, Alexander Sergeev, Arthur Kopylov, Vladimir Rodin, Daniil Malyshev, Tatiana Panova, Igor Polyakov, Maria Zvereva

**Affiliations:** ^1^ Lomonosov Moscow State University, Moscow, Russia; ^2^ Institute of Biomedical Chemistry, Moscow, Russia

**Keywords:** genomics, long-read sequencing, methylomics, Ogataea parapolymorpha DL-1, T2T-assembly

## Abstract

**Background:**

*Ogataea parapolymorpha DL-1* is a versatile thermotolerant organism with numerous applications in biotechnology, particularly in the production of recombinant proteins and the study of methanol metabolism and peroxisome functions. This study presents a comprehensive genome and methylome analysis of *Ogataea parapolymorpha DL-1* using long-read sequencing technology. The research builds upon previous short-read sequencing efforts, revealing enhancements in genome assembly and epigenomic insights.

**Methods:**

We used long-read sequencing technology to achieve a telomere-to-telomere (T2T) genome assembly of *Ogataea parapolymorpha DL-1*. High-quality reads were obtained and assembled *de novo*, followed by polishing to enhance accuracy. The genome was analyzed to identify coding genes, telomeric motifs, rRNA genes, and methylation patterns, including the detection of 5mC and 6 mA modifications. Epigenetic features were further assessed and validated through liquid chromatography-mass spectrometry.

**Results:**

Key findings include the absence of 5 mC DNA modification and the presence of 6 mA in the genome, unusual telomere regulation mechanism based on the addition of non-telomeric dT and the introduction of long-read enhanced telomere-to-telomere assembly.

**Conclusion:**

This work provides deeper insights into the yeast’s genome organization and methylation patterns, contributing to the understanding of its genetics and therefore potential biotechnological applications.

## Introduction

*Ogataea parapolymorpha DL-1* is a methylotrophic yeast with distinctive features and broad applications in both biotechnology and basic research. Known for its capacity to utilize methanol as a sole carbon source, this organism has been extensively studied for its roles in methanol metabolism, peroxisome biogenesis, and related functions. It also serves as a robust cell factory for recombinant protein production and is a promising candidate for metabolic engineering, particularly in high-temperature ethanol production. One of the most intriguing aspects of *Ogataea parapolymorpha DL-1* is its genome organization, particularly in subtelomeric regions, which are enriched with genes involved in diverse metabolic processes. These include transporters for metal ions, amino acids, and carbohydrates, as well as genes associated with redox processes and NADPH regeneration. The dynamic nature of these subtelomeric regions suggests a genetic mechanism for rapid adaptation to environmental changes and stressors (Liebal et al., 2021; Xie et al., 2024).

Transcriptomic analyses reveal that *Ogataea parapolymorpha DL-1* undergoes dramatic gene expression changes when grown on methanol compared to glucose (Ravin et al., 2013). These changes affect various cellular processes, including metabolism, stress responses, and mitochondrial respiratory function. Key upregulated genes include those encoding enzymes for methanol utilization, proteins required for peroxisome assembly and maintenance, and components of the antioxidant defense system. Additionally, phenotype microarray analyses have provided insights into the metabolic versatility of this yeast, highlighting its ability to utilize a wide range of carbon substrates and adapt to diverse growth conditions (Manfrão-Netto et al., 2019).

One of the significant advantages of *Ogataea parapolymorpha DL-1* in biotechnological applications is its highly efficient methanol-inducible promoters, which drive enhanced gene expression in the presence of methanol. Moreover, its derepression in glycerol-containing media offers a competitive advantage over other methylotrophic yeasts, such as *Pichia pastoris*. Another noteworthy trait is its thermotolerance, which facilitates industrial processes by minimizing microbial contamination, reducing cooling costs, and enabling advanced techniques such as Simultaneous Saccharification and Fermentation (SSF) (Ravin et al., 2013).

Telomerase, a ribonucleoprotein enzyme, maintains genomic stability by synthesizing telomeric DNA using an RNA template. In *Ogataea parapolymorpha DL-1*, telomerase uniquely incorporates a non-templated dT nucleotide at telomere ends, contributing to telomere length regulation. This process highlights a distinct telomere maintenance mechanism that differs from other yeasts. Structural analyses of *Ogataea parapolymorpha DL-1* telomerase RNA reveal conserved elements with unique adaptations, emphasizing the enzyme’s evolutionary divergence and functional versatility under thermotolerant conditions (Smekalova ElenaM. et al., 2013).

The yeast has also been a valuable model for telomere biology. Previously referred to as *Hansenula polymorpha DL-1*, strain ATCC 26012 was reclassified as *Ogataea parapolymorpha DL-1* in 2010 (Suh and Zhou, 2010). Its reference genome, assembled in earlier studies, provided few insights into its genomic architecture, subtelomeric regions and repetitive elements (Ravin et al., 2013; Smekalova ElenaM. et al., 2013).

Although previous short-read assembly of *Ogataea parapolymorpha DL-1* was of appropriate quality, it remained incomplete, especially in repetitive and subtelomeric regions critical for functional and structural genomics. We resequenced the genome using advanced long-read sequencing technology to achieve a comprehensive, telomere-to-telomere assembly. T2T assembly would provide a reliable reference for genomic and epigenomic studies and also enable a deeper understanding of telomere biology, genome stability, accurate mapping of DNA methylation states, and serve as a superior benchmark for ongoing comparative genomic and biotechnological research.

DNA methylation is an important epigenetic mechanism influencing gene expression, genome stability, and adaptation (Mattei et al., 2022). In yeasts, 5-methylcytosine (5 mC) has been reported at varying levels, with some species displaying barely detectable amounts of this modification (Tang et al., 2012). Understanding which methylation systems are present or absent helps clarify how these organisms regulate transcription, respond to stress, or maintain genomic integrity. In this study, we specifically investigate 5mC, 5hmC and 6 mA in *Ogataea parapolymorpha DL-1* to determine the exact levels of those modifications.

## Methods

### *Ogataea parapolymorpha DL-1* genomic DNA extraction

*Ogataea parapolymorpha DL-1* yeast was grown in 2 mL of YPD medium (1% (w/v) yeast extract, 2% (w/v) peptone, 2% (w/v) dextrose) at 200 rpm, 37°C overnight. The cells were pelleted by centrifugation at 12,100 × g for 5 min, washed with 500 μ l of deionized water and pelleted again. The pellet was then resuspended in deionized water. Next, 200 μ l of a phenol:chloroform:isoamyl alcohol mixture in a ratio of 25:24:1, 0.3 g of glass beads, and 200 μ l of YLB buffer (10 mM Tris-HCl, 1 mM EDTA, 2% Triton X-100, 1% SDS, pH 8.0) were added. The mixture was vortexed for 2 min, diluted with 200 μ L of TE buffer (10 mM Tris-HCl, 1 mM EDTA, pH 8.0) and centrifuged at 14,000 rpm for 5 min. The aqueous phase was collected and diluted with 1 mL of 96% ethanol. After centrifugation at 14,000 rpm for 5 min, the supernatant was collected, and the pellet was dissolved in 400 μ l of TE buffer. Then, 3 μ l of ribonuclease A (10 mg/mL) was added, and the mixture was incubated at 37°C for 5 min 10 μ l of 4 M NH4OAc and 1 mL of ethanol were then added to the mixture. After centrifugation at 14,000 rpm for 5 min, the supernatant was collected. The pellet was dried at 45°C and dissolved in 30 μ l of TE buffer. The concentration of the isolated DNA was 1.1 μ g/ μ l. The quantity and quality of the extracted DNA were controlled by migration on agarose gel (Supplementary Figure S1), spectrophotometry (Nano-500 spectrophotometer, Allsheng, China), and fluorometric quantification (Qubit, ThermoFisher Scientific, MA, United States).

### Library preparation

PromethION sequencing libraries were prepared using 1 μ g of *Ogataea parapolymorpha DL-1* genomic DNA according to the SQK-NBD114.24 Ligation Sequencing gDNA - Native barcoding kit 24 V14 (PromethION) protocol.

### PromethION flow cell preparation and DNA sequencing

The sequencing mix was prepared with 35 μ l of the barcoded DNA library according to the SQK-NBD114.24 Ligation Sequencing gDNA - Native barcoding kit 24 V14 (PromethION) protocol. The mix was added to PromethION R10.4.1 flow cell loaded into P2 Solo sequencing unit (Oxford Nanopore, Oxford, UK). The sequencing run lasted 1 h and 21 min yielding 844 K raw reads and 2.6 Gb of sequence data in POD5 format. MinKNOW software (version 5.7.2) was used to initialize and monitor the sequencing run.

### Raw sequencing data processing

Dorado (Oxford Nanopore Technologies, 0.9.0+9dc15a8, model dna_r10.4.1_e8.2_400bps_sup@v4.3.0), was used for basecalling and demultiplexing. Porechop (v0.2.4) (Bonenfant et al., 2022) was used for adapter trimming. FastQC (v0.12.1) (FastQC, 2024) was used to assess the quality of reads. Fastp (v0.24.0) (Chen et al., 2018) was used to filter out low quality reads with a threshold of Q35.

### *De novo* genome assembly and polishing

Long-read genome assembly was performed using miniasm (v0.3) (Li, 2016), a fast and lightweight assembler that constructs contigs based on read overlaps without performing error correction. Filtered sequencing reads were used to identify overlaps using minimap2 (v2.24-r1122) (Li, 2018) with the “ava-ont” preset for all-vs-all read alignment. The resulting overlap file in PAF format was then used as input for miniasm using default parameters for assembly. The assembly graph was generated in GFA format and subsequently converted to FASTA using a custom awk script. Since miniasm does not perform consensus error correction, the assembled sequences were further polished using medaka (v2.0.1, model r1041_e82_400bps_sup_v4.3.0, Oxford Nanopore Technologies), a neural network-based tool specifically designed for Oxford Nanopore data. Raw reads were aligned to the draft assembly using minimap2, and medaka_consensus was run with default parameters. The final polished assembly was used for downstream analysis.

### Quality evaluation and genomic features annotation

Quality of *De novo* assembled genome was evaluated using two reference free quality assessment tools Inspector (v1.3) (Chen et al., 2021) and Merqury (v1.3) (Rhie et al., 2020). To generate a k-mer database for reference-free quality assessment using Merqury, we used Meryl tool (v.1.3, part of Merqury) to count 21-mers from high-accuracy short-read sequencing data. Coding genes were annotated with AUGUSTUS (v3.5.0) (Stanke and Augustus, 2005). rRNA genes were found with rRNAFinder (v1.1.1) (Dong, 2019). Comparison with previous assembly was carried out by the use of MUMmer (v4.0.1) (Marçais et al., 2018). Assembly to assembly alignment was visualized with CIRCOS (v0.52) (Krzywinski et al., 2009). Tandem repeats were annotated with RPTRF (v1.0) (Behboudi et al., 2023).

### Methylation analysis

5mC, 5hmC and 6 mA modification data was called from raw pod5 data using dorado basecaller (Oxford Nanopore Technologies, version 0.9.0+9dc15a8, models dna_r10.4.1_e8.2_400bps_sup@v4.3.0_5mC_5hmC@v1 and dna_r10.4.1_e8.2_400bps_sup@v4.3.0_6mA@v2), bedmethyl files were obtained using modkit pileup (Oxford Nanopore Technologies, version 0.4.1). The threshold for counting modifications was frequency >0.5 and coverage >10X.

To assess the consistency of the modification calling results, we took a ‘hac’ model with all of the versions available (hac_v4.3.0_6 mA_v1, hac_v4.3.0_6 mA_v2, hac_v5.0.0_6 mA_v1, hac_v5.0.0_6 mA_v2, hac_v5.0.0_6 mA_v3, hac_v4.3.0_5 mC_5hmC_v1, hac_v5.0.0_5 mC_5hmC_v2, hac_v5.0.0_5 mC_5hmC_v3. Supplementary Table S2; Supplementary Table S3; Supplementary Table S4).

Methylation motifs were searched using MEME (v5.5.7) (Bailey and Elkan, 1994). To extract a batch of sequences for MEME we searched for coordinates of modified bases with high frequency >0.5 and coverage >10X, then added 10 bp upstream and downstream those coordinates resulting in a bed file of 21 bp-long sequences. Fasta file was created using bedtools (v2.31.1) (Quinlan and Hall, 2010).

To validate the absence of 5 mC in our primary study organism, we performed a parallel sequencing analysis of the *Escherichia coli* genome as a positive control.

Methylation density over chromosomes was calculated as follows:

$$\text{Methylation Density}=\frac{N_{\text{mod}}}{N_{\text{mod}}+N_{\text{canonical}}}÷{\text{chr}}_{i,\text{total length}}$$ (1)

Methylation density per 1 Kbp in the specific chromosome was calculated as follows:

$$\text{Methylation Density}=\frac{N_{\text{mod}}}{N_{\text{mod}}+N_{\text{canonical}}}÷1000$$ (2)

### Search for putative methyltransferases in *Ogatea parapolymorpha DL-1* genome

To find putative methyltransferases we created a reference database of protein sequences fasta files using UniProtKB (The UniProt Consortium, 2016) with the query: ““DNA methyltransferase” AND ((N6-adenine) OR (cytosine-5)) NOT (RNA OR tRNA)”. It yielded 121,392 results. We used DIAMOND (Buchfink et al., 2021) (v 2.1.11) to create a database out of UniProtKB’s search results. We then took protein sequences predicted by AUGUSTUS and searched them over a constructed database using diamond blastp in sensitive mode with default parameters. Protein sequences from top 5 search results were subjected to online BLAST (Altschul et al., 1990) search to NCBI’s non-redundant (NR) protein database.

### LC-SRM analysis and instrumentation

Samples were analyzed on an Infinity II 1290 UPLC system (Agilent, Santa Clara, CA, United States) hyphenated with a G6490A triple quadrupole mass spectrometer (Agilent, Santa Clara, CA, United States). The UPLC system was equipped with an Acquity HSS T3 column (150 × 2.1 mm, 1.8 μ m particle size; Waters, Milford, MA, United States). The column was heated at 40°C. Ten μ L of sample was loaded in the column and separated at 0.2 mL/min in gradient of mobile phase A (water) and mobile phase B (60% acetonitrile in water) both supplied by 0.1% formic acid and fortified by 0.02% HFIP. The following gradient of elution was applied: initial condition – 1% of B; from 0 to 2.5 min–4.8% of B; increasing of B to 28% at 17 min; increasing of B to 46% at 24 min; rapid increase of B to 98% at 26 min; isocratic washing in 98% of B for 4 min; returning to initial condition of B at 31 min; equilibrating the column in initial gradient conditions for the next 3 min.

The mass spectrometer was equipped with a Jet Stream ionization source and operated in a positive ionization mode with a drying gas (nitrogen) temperature of 260 °C at a flow rate of 13 L/min and sheath gas (nitrogen) temperature of 320 °C at a flow rate of 7 L/min. The capillary voltage was set to 3200 V and the nozzle voltage was set to 600 V with a high pressure RF adjusted to 120 V and low pressure RF to 55 V. Spectra were acquired in SRM (selected reactions monitoring) mode for the duty cycle time of 238.5 milliseconds (4.19 cycles per s). Nucleotides were detected by transitions using an optimized set of collision energy, cell accelerating voltage, and dwell time parameters (Table 1). Reference standards: 2′-Deoxyadenosine, 2′-deoxycytidine, 2′-deoxyguanosine (Sigma, Germany), 5-(methyl-d3)-2′-deoxycytidine (Santa Cruz Biotechnology, TX, United States).

**TABLE 1:** SRM parameters optimized for acquisition of nucleotides.

Compound	dG	dA	dC	dT	d5mC	d6mA
Precursor ion m/z	268.1	252.1	228.1	243.1	242.1	265.1
Fragment ion m/z	151.9	136.1	112.1	127.1	126.1	150.3
Collision energy, eV	20	20	18	17	19	25
Cell accelerating voltage, V	3	3	2	2	2	3
Retention time, min	6.88	6.18	4.97	9.86	5.05	10.27
Dwell time, milliseconds	80	55	60	80	40	55

External standard (ESTD) was adulterated by combing of nucleotide standards and diluted in 0.1% formic acid to 100 pg/ μ L finally.

### Dot blot 6 mA analysis

Genomic DNA from *Ogataea parapolymorpha DL-1* was isolated and treated with Proteinase K (overnight, 40°C in Q5 buffer) to remove residual proteins, followed by heat inactivation at 95°C and purification using magnetic beads (GentaPure kit). DNA was denatured by incubation with 2 M NaOH and 100 mM EDTA at 95°C for 10 min, chilled on ice, and neutralized with 1 M Tris-HCl (pH 7.5).

Denatured DNA (3 μ L per spot) was applied to nitrocellulose membranes, UV-crosslinked (125 mJ/cm^2^, 3 × ), and blocked in TBST with 5% BSA. Membranes were incubated overnight at 4°C with anti-6mA antibodies (1:5,000), followed by HRP-conjugated secondary antibodies (1:5,000, 1 h, room temperature). Chemiluminescent detection was performed using the EasySee Western blot Kit (TransGen Biotech), and signals were imaged with a Chemidoc Imaging System (Bio-Rad).

DNA from *Raji* cells and *Escherichia coli* served as positive controls for 6mA, while unmethylated PCR products were used as a negative control (Supplementary Figure S2).

### 3′-telomere extension

To investigate the addition of non-telomeric nucleotides at telomeric ends, we processed BAM files containing reads aligned to defined telomeric regions across all chromosomes. Quality trimming of reads was applied conservatively (bases with quality scores below Q30 were removed) to eliminate ambiguous or low-confidence base calls at the read termini. Quality trimming at Q30 ensures that the terminal nucleotide retained in each read is confidently sequenced and reliably represents the true biological end of the read, rather than a sequencing artifact or low-confidence call. Bases removed during trimming were of low confidence and, therefore, likely erroneous; bases remaining after trimming are thus of high-quality, robust indicators of actual genomic termini and suitable for accurate biological interpretation. Subsequently, we isolated the last 200 base pairs from the trimmed reads and specifically quantified occurrences at the ends of trimmed reads of thymine (T), as well as adenine (A) as its complementary base. By calculating the percentage of trimmed reads ending with these nucleotides, we confidently assessed the prevalence of non-templated nucleotide additions at telomere ends, ensuring that detected terminal nucleotides represent genuine genomic termini rather than sequencing artifacts.

## Results

The resulting total amount of reads after fastp filtration with a quality threshold of Q35 (>80% of bases with Q35) was 285,134 with an average read length of 3,402 bp (Table 2). The threshold for quality was chosen as Q35 in order to avoid hybrid assembly with its obligation of obtaining high-quality short reads. After aligning reads to a previously reported assembly (PRJNA60503), the resulting coverage of *Ogataea parapolymorpha DL-1* was 102X, and the coverage of the positive control *E. coli* was 112X. There were no plasmids assembled *de novo* from the reads, only seven chromosomes and one circular mito genome.

**TABLE 2:** Descriptive statistics of sequencing data before and after filtration.

	N50	N reads	Gb
Raw	11,044	761,276	2.42
After filtration	11,915	285,134	0.97

Long-read genomic assembly reveals an extension in the length of every chromosome compared to previous data. (Table 3).

**TABLE 3:** Comparison of long-read and short-read assemblies with the corresponding delta percentages.

Contig_Name	Long-read assembly	Short-read assembly	Delta, %
chr1	990,600	955,766	3.645
chr2	994,175	990,963	0.324
chr3	1,276,591	1,273,462	0.246
chr4	1,299,983	1,293,628	0.491
chr5	1,332,873	1,330,267	0.196
chr6	1,517,422	1,514,933	0.164
chr7	1,515,586	1,515,570	0.001
mitochondrion	41,721	41,719	0.005

As shown in Table 4, the QV scores calculated with Mercury and Inspector for the long-read-based assembly of *Ogataea parapolymorpha DL-1* were consistently higher than those for the short-read-based draft. This confirms that the long-read assembly provides a more accurate representation of the genome. For additional validation, we evaluated an *E. coli* K-12 BW25113 delta-MutM reference assembly as a control. Both tools reported high QV scores for *E. coli*, consistent with its well-characterized, high-quality reference, further supporting the reliability of the evaluation metrics.

**TABLE 4:** Comparison of QV scores reported by Inspector and Merqury.

Tool	Long-read assembly (DL-1)	Short-read assembly (DL-1)	*E. coli* References
Inspector	48.45	37.02	49.51
Merqury	55.75	48.83	50.75

Our long-read based assembly achieves a telomere-to-telomere (T2T) completeness, a significant improvement over the previous assembly that only identified incomplete parts of telomeric sequences for chromosomes 4 and 7. Our analysis has confirmed the presence of the GGTGGCGG telomere motif (Table 5). Furthermore, the telomeres in our assembly are considerably longer, by 2–3 times, than those noted in the earlier assembly for chromosomes 4 and 7. In contrast to this incomplete representation, our gapless T2T assembly provides a seamless genomic map (Figure 3). The breakpoints identified in the assembly-to-assembly alignment coincided precisely with the gaps previously reported in chromosomes 1 and 4 of the earlier assembly.

**TABLE 5:** Telomeric sequences found in the long read and short read assemblies.

Name	Start	End	Length, bp	Motif	Feature	Assembly
chr1	0	179	177	GGTGGCGG	Telomere_5_prime	long (T2T)
chr2	0	141	138		Telomere_5_prime	long (T2T)
chr3	0	158	155		Telomere_5_prime	long (T2T)
chr4	0	124	123		Telomere_5_prime	long (T2T)
chr5	0	191	141		Telomere_5_prime	long (T2T)
chr6	0	193	103		Telomere_5_prime	long (T2T)
chr7	0	111	110		Telomere_5_prime	long (T2T)
chr1	990,461	990,594	134		Telomere_3_prime	long (T2T)
chr2	994,069	994,173	105		Telomere_3_prime	long (T2T)
chr3	1,276,403	1,276,589	187		Telomere_3_prime	long (T2T)
chr4	1,299,816	1,299,982	166		Telomere_3_prime	long (T2T)
chr5	1,332,697	1,332,871	175		Telomere_3_prime	long (T2T)
chr6	1,517,270	1,517,420	151		Telomere_3_prime	long (T2T)
chr7	1,515,437	1,515,526	90		Telomere_3_prime	long (T2T)

**FIGURE 1:**
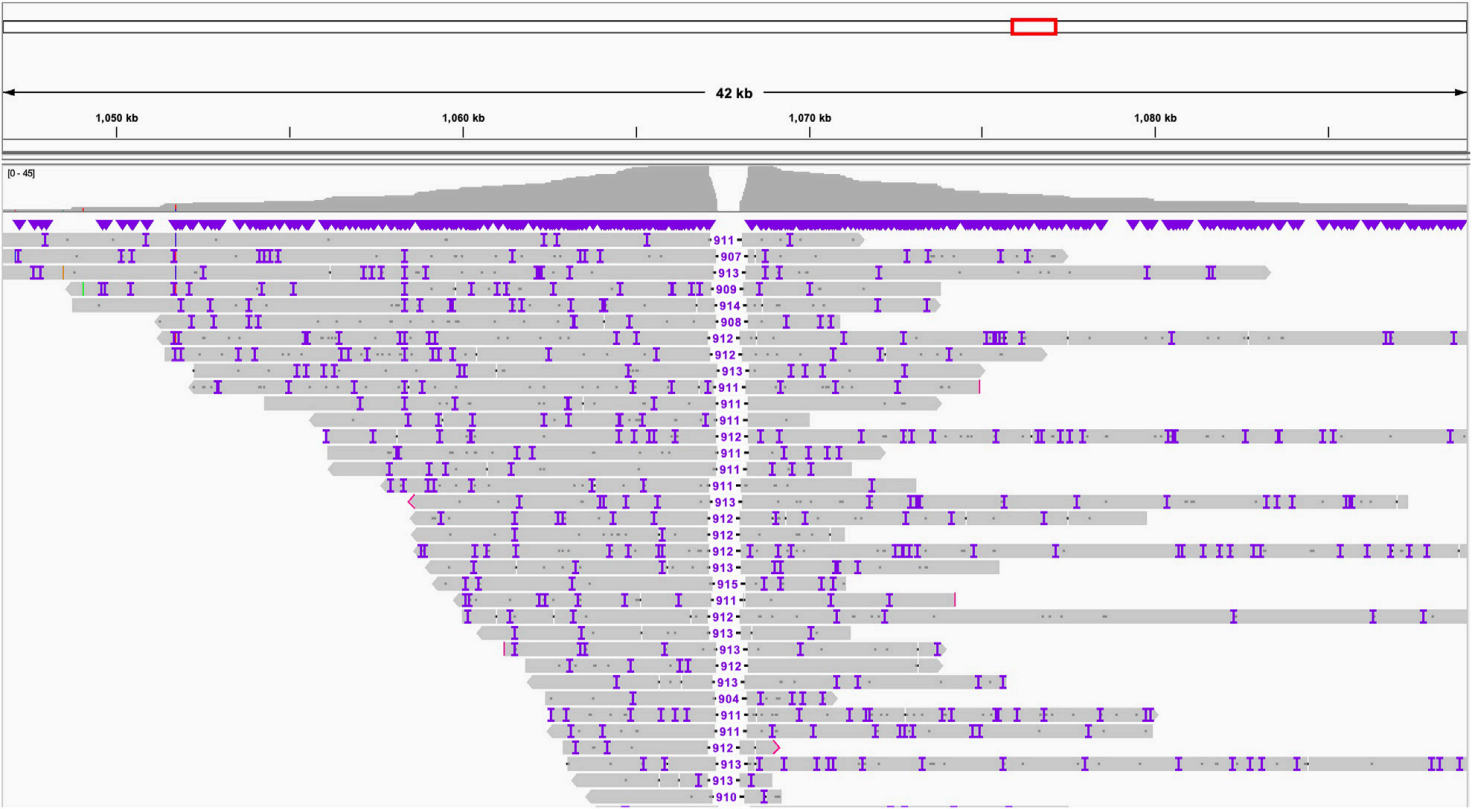
Expansion chr7:1,067,235-1,068,147 in the old assembly revealed by Inspector using long-read data.

Through the application of “Inspector” (Chen et al., 2021) a tool for reference-free assembly quality assessment, we detected an erroneous expansion within chromosome 7 of up to 1,000 bp in the previous short-read assembly, highlighting the superior resolution of long-read sequencing in identifying and correcting assembly errors (Figure 1).

Examination of rRNA gene encoding regions has showed the presence of five copies of each of the four types of rRNA genes (Table 6), in contrast to the single copy of three genes found in the previous assembly.

**TABLE 6:** rRNA genes coordinates in the newly assembled genome.

Chromosome	Gene type	Start	End	Strand
chr1	euk_18SrRNA	310,756	312,546	-
chr1	euk_28SrRNA	306,752	310,102	-
chr1	euk_5SrRNA	297,582	297,699	+
chr1	euk_5_8SrRNA	326,556	326,707	-
chr1	euk_18SrRNA	335,142	336,932	-
chr1	euk_5SrRNA	321,968	322,085	+
chr1	euk_28SrRNA	323,009	326,359	-
chr1	euk_5_8SrRNA	310,299	310,450	-
chr1	euk_5SrRNA	313,840	313,957	+
chr1	euk_5SrRNA	330,097	330,214	+
chr1	euk_28SrRNA	298,623	301,973	-
chr1	euk_5_8SrRNA	318,427	318,578	-
chr1	euk_5_8SrRNA	334,685	334,836	-
chr1	euk_5_8SrRNA	302,170	302,321	-
chr1	euk_18SrRNA	318,884	320,674	-
chr1	euk_18SrRNA	327,013	328,803	-
chr1	euk_28SrRNA	331,138	334,488	-
chr1	euk_28SrRNA	314,880	318,230	-
chr1	euk_5SrRNA	305,711	305,828	+
chr1	euk_18SrRNA	302,627	304,417	-

Analysis of the telomeric regions across various chromosomes of Ogataea parapolymorpha DL-1 revealed a significant predominance of thymine (T) nucleotides at the 3′ ends of the telomeres, supporting the hypothesis of non-telomeric dT addition by telomerase (Table 7 “A_end” - number of Adenines at the 3′end, “T_end” -- number of Thymidines respectively, “perc_A” - percentage of 3′end aligned reads containing dA at the end. N_total_reads - total reads, aligned to the 3′end).

**TABLE 7:** Count of nontelomeric dT addition derived from reads aligned to the 3′end of the chromosomes.

Region	A_end	T_end	perc_A	perc_T	N_total_reads
chr1:990,461-990594	3	37	5	64	58
chr2:994,069-994173	3	78	3	73	107
chr3:1,276,403-1276589	6	117	4	75	155
chr4:1,299,816-1299982	13	126	7	71	178
chr5:1,332,697-1332871	6	107	4	72	148
chr6:1,517,270-1517420	4	68	4	72	94
chr7:1,515,437-1515541	10	69	9	60	115

The respective counts of basecalled modifications for Ogataea parapolymorpha DL-1 and positive control *Escherichia coli* are presented in the (Table 8). Based on modification calling data, the mitochondrial genome, as well as the other chromosomes, had the 6 mA modifications and did not have the 5 mC.

**TABLE 8:** Comparative analysis of total number modifications (5mC, 5hmC and 6 mA) found with dorado. Percentage in brackets shows the part from all of the A or C in the genome.

	5 mC	5hmC	6 mA
*Ogataea parapolymorpha DL-1*	123 (0.0057%)	614 (0.029%)	21,050 (0.91%)
*Escherichia coli*	24,181 (1.68%)	1,669 (0.12%)	82,863 (5.92%)

**FIGURE 2:**
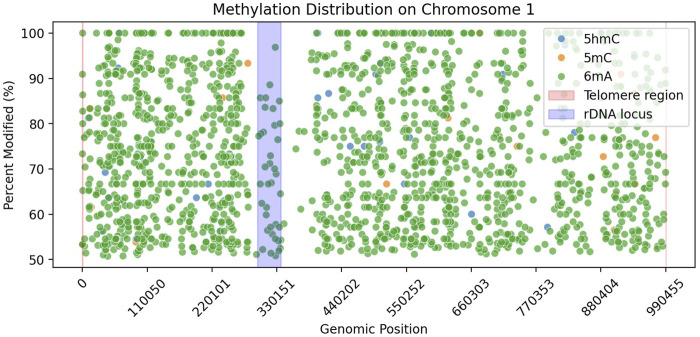
Methylation distribution along Chromosome 1 of *Ogataea parapolymorpha DL-1*. Scatter plot shows genomic positions versus percent modified sites for three DNA modifications: 5hmC (blue), 5 mC (orange), and 6 mA (green), filtered by >50% modification and >10 × coverage. Shaded regions highlight telomeric ends (red) and the rDNA locus (blue) on the chromosome.

We explored the little amount of 5 mC data that was basecalled. We looked at the rDNA locus (Figure 2) to check whether it contains 5 mC or not, as it was previously reported to be present in other yeast species (Sandesh Pai et al., 2022). Our data showed no 5 mC in the rDNA locus of *Ogataea parapolymorpha DL-1*.

To validate the absence of 5mC, genomic DNA samples isolated from the *Ogataea parapolymorpha DL-1* yeast were hydrolyzed to the nucleosides, and the content of 5 mC was determined using UPLC/MS-MS. 5mC was not detected in the hydrolyzed samples, confirming the results obtained from nanopore sequencing data (Supplementary Table S1).

The presence of 6 mA in *Ogataea parapolymorpha DL-1* genomic DNA was confirmed by dot blot analysis (Supplementary Figure S2).

Sequence motif TCCACCA was found in the regions ± 10 bp from 6 mA sites in *Ogataea parapolymorpha DL-1*. The site itself wasn’t methylated suggesting that it is used by methyltransferases to modify adenines nearby. In the validation experiment with *E. coli* known motifs GmATC and CmC(T/A)GG were found as previously reported (Kahramanoglou et al., 2012) (Table 9).

**TABLE 9:** Modifications analysis. Motifs found with MEME in regions ± 10 bp from methylated sites.

	Sequence of motif	Number of motifs in regions ± 10 bp from methylated sites	E-Value
*Ogataea parapolymorpha DL-1*	TCCACCA	875	4.2e-021
*Escherichia coli*	GmATC	41,833	1.2e-384
*Escherichia coli*	CmC(T/A)GG	21,357	1.9e-1156

**FIGURE 3:**
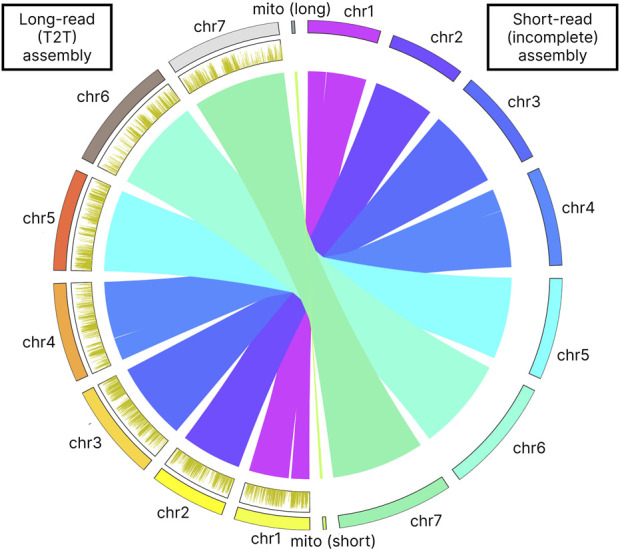
Assembly-to-assembly alignment visualization. The figure also shows the 6 mA methylation profile on the left side of the figure as distribution plot. 6 mA density plots were computed in 1 kb bins.

Methylation density analysis showed a uniform distribution across the chromosomes. Density per 1 Kbp also was consistent across the chromosomes showing approximately 2 6 mA modifications per kilobase (Table 10 Figure 3).

**TABLE 10:** 6 mA Density per chromosome and per 1Kbp for different chromosomes calculated using Equation 1 and Equation 2.

	6 mA density per chromosome	6 mA density per 1Kbp
chr1	0.0024	2.3687
chr2	0.0021	1.9862
chr3	0.0023	2.8851
chr4	0.0017	2.1590
chr5	0.0020	2.7048
chr6	0.0014	2.1902
chr7	0.0013	1.9909
mitochondrion	0.0004	0.0172

Search for putative methyltransferases in *Ogatea parapolymorpha DL-1* genome with diamond blastp reported 279 partial alignments. No homologous methyltransferases were found using this method.

## Discussion

### Long read based enhanced WGS assembly

As we produced a huge amount of high quality sequencing data, we were able to use only long reads for assembly and do not produce short reads for the hybrid approach. For samples with an average read length of 8 Kbp or more, a coverage depth of 30X is sufficient (Khrenova et al., 2022). For samples with shorter reads, averaging around 2 Kbp, a higher coverage depth of 50X is required. Our reads with N50 more than 11 Kbp and coverage depth more than 100X allowed for high quality genomic assembly. We also filtered data to ensure highest quality. Our analysis of *Ogataea parapolymorpha DL-1* has culminated in the acquisition of extended chromosome lengths compared to the preceding short-read assembly. Specifically, chromosome 1 exhibited the most significant extension, growing from 955,766 base pairs in the short-read assembly to 990,600 base pairs in the long-read assembly, marking a delta of 3.645%. Similarly, chromosome 4 saw a noteworthy lengthening, from 1,293,628 to 1,299,983 base pairs, reflecting a 0.491% increase. What is more, those two chromosomes were the only ones that could not be assembled gaplessly in the previous study.

Both tools Inspector and Merqury were used to evaluate the consensus quality (QV) of the genome assemblies in a reference-free manner. These tools rely on distinct principles and provide complementary insights. Inspector evaluates assembly quality by aligning long reads back to the assembly and identifying small-scale and structural errors directly from read-to-contig alignments. It is particularly well-suited for identifying localized misassemblies and quantifying structural integrity, including inversions, collapses, and haplotype switches. Merqury, on the other hand, assesses base-level consensus accuracy through k-mer spectra analysis. It calculates consensus Quality Value (QV), completeness, and haplotype phasing based on the presence or absence of k-mers in the assembly compared to the read set. In our study, both tools showed broadly consistent results (Table 4) but differed in absolute QV values, which can be attributed to their underlying methodologies.

We also analyzed the rDNA locus within chromosome 1. The rDNA locus in most yeast species, including *Ogataea polymorpha DL-1*, consists of tens or hundreds of tandemly repeated units, typically spanning hundreds of kilobases to over a megabase in length (Kobayashi and Sasaki, 2017). Genome assemblies generally represent this array as a collapsed segment due to the repetitive nature of the region. In our study, we identified five tandemly repeated units of the rDNA locus in our assembly, including complete copies of the 18S, 28S, 5S, and 5.8S rRNA genes. However, this does not reflect the total number of rDNA repeats in *Ogataea polymorpha DL-1*, as determining the full array size would require extremely long sequencing reads (approximately 1 Mb or longer) that traverse the entire rDNA array. A comparative analysis between the two assemblies of chromosome 1 revealed a substantial deletion of 32,415 base pairs in the previous short-read assembly. This deleted region corresponds to the rDNA locus and includes tandemly repeated units of the 18S, 28S, 5S, and 5.8S rRNA genes. This specific nature of genomic sequence is the most probable reason why the previous short-read based assembly had a gap in this region. Unfortunately, its not clear why the chromosome 4 and 7 had misassembly. We examined those regions for tandem repeats using RPTRF and did not find any repeats spanning longer regions than short reads are able to cover.

Using the Inspector tool, we uncovered another misassembled region in previously reported draft - a significant expansion in chromosome 7 (Figure 1). This expansion, spanning 912 base pairs (from position 1,067,235 to 1,068,147), represents a notable deviation from the newly established long-read assembly. Though it is not clear why did the previous effort this region.

Our analyses also confirmed the presence of the previously identified telomeric motif (GGTGGCGG) at the 3′ ends. This confirmation across all chromosome ends, coupled with the discovery of the 5′ CCACCCCG motif, enhances our understanding of the chromosomal termini in this species. The detailed data, including coordinates and lengths of these telomeric regions, are provided in the accompanying table. In contrast to the previous short-read assembly of *Ogataea parapolymorpha DL-1*, our long-read sequencing approach successfully closed the gaps, resulting in a complete and continuous genome. Specifically, the previous assembly exhibited gaps in chromosomes 1 and 4, at positions chr1:297,310–297409, chr1:305,147-305246, and chr4:366,734-370733.

### 3′-telomere extension

In the study (Smekalova EM. et al., 2013) of telomerase activity within *Ogataea parapolymorpha DL-1*, a remarkable discovery was made regarding the addition of a non-telomeric dT nucleotide by the telomerase enzyme. Unlike the conventional understanding of telomerase function, which primarily focuses on the elongation of telomeres through the addition of specific telomeric repeats to counteract telomere shortening, *Ogataea parapolymorpha DL-1* exhibits a unique mechanism of telomere length regulation in budding yeasts (Malyavko et al., 2014). The telomerase RNA from this thermotolerant yeast species, identified as HpTER, extends beyond merely synthesizing telomeric sequences. It actively incorporates a dT nucleotide beyond the anticipated boundary of the RNA template *in vitro*, a modification not part of the telomeric repeat itself. Subsequent sequencing of chromosomal ends carried out by the authors (Smekalova EM. et al., 2013) validated the presence of this dT nucleotide as a terminal element at the 3′ end of telomeres with independent methodological confirmation of the presence of an additional nontelomeric nucleotide at the 3′-end of the chromosomes (Malyavko et al., 2016). Mutational analysis of HpTER template region elucidated that this unconventional addition of non telomeric nucleotide plays a crucial role in limiting telomere length within *Ogataea parapolymorpha DL-1* and one of natural mechanism of ‘chromosome capping’ for telomere length regulation (Wellinger, 2010).

In our study we have confirmed this finding using long read sequencing technology. This phenomenon underscores a novel layer of complexity in telomere length regulation, revealing that telomerase can extend its functional repertoire beyond traditional repeat addition. This adaptation suggests a species-specific regulatory mechanism for telomere maintenance, potentially contributing to the genomic stability of *Ogataea parapolymorpha DL-1* and highlighting the diversity of telomerase activity across different organisms.

### DNA methylation anomaly: Absence of 5 mC

A paramount discovery is the absence of 5-methylcytosine (5 mC), which was supported by the UPLC data. This suggests an evolutionary trajectory where the organism has possibly discarded the methylation system typically essential for other species, yet maintains normal physiological functions. The dorado modification calling model is prone to producing errors, nevertheless, in Table 8 we see that *Ogataea parapolymorpha DL-1* doesn’t have the cytosine modification if we compare it to *E. coli* which does have it. We also observe that the count of 5hmC for *Ogataea parapolymorpha DL-1* exceeds the count of 5mC, which is impossible because 5hmC is produced from 5 mC. It should also be mentioned that different modification calling tools and different versions of models for them are prone to producing different results. For example, it was reported that the open-source 5 mC analysis tool DeepMod 2 (Umair Ahsan et al., 2024) demonstrated greater accuracy in detecting canonical cytosines compared to Oxford Nanopore’s Dorado in study (Sergeev et al., 2025). In our study, we have shown the inconsistency of modification calling results by comparing different models for dorado basecaller (Supplementary Table S2; Supplementary Table S3; Supplementary Table S4). This result clearly shows that the modification data obtained from nanopore sequencer must be confirmed by orthogonal methods such as LC-SRM or dot blot used in our study.

Positive control *E. coli* in contrast, shows that the number of 5 mC exceeds 5hmC greatly. This suggests that the minimal amount of modified cytosines basecalled for Ogataea parapolymorpha DL-1 is attributable to errors in the basecalling model. The phenomenon of DNA methylation, a crucial epigenetic mechanism for regulating gene expression, exhibits remarkable variability across other living organisms like the fungal kingdom, primarily due to the differential presence of DNA methyltransferases (DNMTs) (Nai et al., 2021). In certain fungal species, the complete absence of DNMTs shows the lack of DNA methylation, highlighting a direct link between the availability of these enzymes and the existence of methylation processes within the genome. This absence indicates that some fungi might employ alternative epigenetic strategies for gene regulation, diverging significantly from the methylation-dependent pathways observed in other eukaryotes. There are other reported cases in which 6 mA is present and 5 mC is not present in the genome (Lieberman Greer et al., 2015), (Lizarraga et al., 2020).

In our study, we observed an absence of detectable 5 mC in *Ogataea parapolymorpha DL-1*, consistent with previous findings suggesting that DNA methylation in budding yeast species can vary significantly, often presenting at low or undetectable levels. For instance, low-level methylation (0.014%–0.364%) has been reported in various budding yeast species including *Saccharomyces cerevisiae* (Tang et al., 2012), often only detectable by highly sensitive analytical methods such as GC/MS. In contrast, our analysis utilizing nanopore sequencing complemented by ultra-sensitive UPLC-MS/MS did not detect 5 mC modifications. Additionally, the few nanopore-based predictions of 5 mC we identified (Table 8) were closely scrutinized and attributed to algorithmic false positives due to their extremely low frequency and lack of reproducibility by orthogonal methods. Furthermore, the genomic distribution of these few computationally predicted 5 mC sites did not align with previously reported methylation hotspots such as the rDNA locus (Figure 2) observed in *Saccharomyces cerevisiae* (Sandesh Pai et al., 2022).

Given these observations, we conclude that the absence of 5 mC in *Ogataea parapolymorpha DL-1* is authentic rather than a limitation of analytical sensitivity, and it may reflect evolutionary divergence in epigenetic regulatory mechanisms among closely related yeast species.

### Possible methyltransferase motif

In our study, we identified a potential methyltransferase-associated motif, TCCACCA (Table 9), within the Ogataea parapolymorpha DL-1 genome. Interestingly, 6 mA is not located within the site. Instead, the modified base could be found up to 4 base pairs upstream of this site.

It is known from other organisms that methylation motifs do not always directly coincide with the modified bases (Wang et al., 2019). Rather, these motifs commonly serve as binding sites recognized by methyltransferases or associated proteins, thereby influencing methylation states at adjacent nucleotides. For instance, in bacteria such as *Escherichia coli*, the known DNA methyltransferase Dam recognizes and methylates adenine residues within a specific GATC motif, directly methylating adenines within that precise context (Kahramanoglou et al., 2012). However, in contrast, studies in eukaryotes, such as *Tetrahymena thermophila* and *Chlamydomonas reinhardtii*, have shown that 6 mA methyltransferases may recognize extended sequence contexts or distal motifs rather than direct, contiguous recognition sequences (Cheng et al., 2019).

To our knowledge, no data currently exist on this specific TCCACCA motif or similar motifs in *Ogataea parapolymorpha DL-1* or other closely related yeast species. Additionally, no characterized methyltransferase in yeast has been linked to the recognition of this sequence.

### Conclusion

Our comprehensive investigation into the *Ogataea parapolymorpha DL-1* genome through long-read sequencing technology has advanced the understanding of this organism’s genomic architecture and methylation patterns. By achieving a telomere-to-telomere assembly, we have closed previous gaps and also extended the known lengths of chromosomes, offering a complete and continuous genomic map. This study has also uncovered crucial genomic regions previously unresolved, including a significant 32,415 bp deletion containing multiple rRNA gene copies, enhancing our insight into the genomic organization and its functional implications in rRNA processing. The absence of 5-methylcytosine across the genome suggests unique methylation dynamics and an evolutionary divergence from typical eukaryotic methylation patterns. These findings underscore the power of long-read sequencing to reveal detailed and accurate genomic features that are often obscured by short-read sequencing technologies. This work enriches our genomic knowledge of *Ogataea parapolymorpha DL-1* and contributes to the broader understanding of genomic structure and stability in eukaryotes, paving the way for future studies on genomic and epigenetic diversity.

## Data Availability

The Genomic assembly as well as the sequencing reads generated in this study have been submitted to the NCBI BioProject database under accession number PRJNA1091144.
