# Supplementary material for: Long-read sequencing reveals absence of 5mC in Ogataea parapolymorpha DL-1 genome and introduces telomere-to-telomere assembly
Source: Front Genet. 2025 May 9;16:1574332. doi: 10.3389/fgene.2025.1574332 (PMC12098377; doi:10.3389/fgene.2025.1574332)
Supplement: Supplementary file 2 [file Table1.docx]

**Supplementary Materials**

**Table S1.** SRM data for the identification of 5-methylcytidine in Ogataea parapolymorpha DL-1 genome

| General | | | | | | | | | Compound  Identification | | Area Average | Concentration in ESTD, pg/mL | Concentration in Sample, pg/mL |
| --- | --- | --- | --- | --- | --- | --- | --- | --- | --- | --- | --- | --- | --- |
| Label | RT | Width | Height | Area | Score | Mining Algorithm | CE | m/z | Cpd | Name |  |  |  |
| Cpd 1: dm5C 5.268 | 5,268 | 0,32 | 149 | 896 | 100 | Find by MRM | 18 | 242,1 | 1 | dm5C ESTD | 964,3 | 100±5,23 pg/uL | - |
| Cpd 1: dm5C 5.076 | 5,076 | 0,365 | 162 | 960 | 100 | Find by MRM | 18 | 242,1 | 1 |  |  |  |  |
| Cpd 1: dm5C 5.058 | 5,058 | 0,408 | 176 | 1013 | 100 | Find by MRM | 18 | 242,1 | 1 |  |  |  |  |
| Cpd 1: dm5C 5.045 | 5,045 | 0,368 | 156 | 988 | 100 | Find by MRM | 18 | 242,1 | 1 |  |  |  |  |
| Cpd 3: dm5C | - | - | - | - | 100 | Find by MRM | 18 | 242,1 | 3 | dm5C Sample | **-** | **-** | **0,00**  **pg/uL** |
| Cpd 3: dm5C | - | - | - | - | 100 | Find by MRM | 18 | 242,1 | 3 |  |  |  |  |
| Cpd 4: dC 4.973 | 4,973 | 0,244 | 11 | 61 | 100 | Find by MRM | 18 | 228,1 | 4 | dC Sample | 62,0 | - | **6,43±0,023 pg/uL** |
| Cpd 4: dC 3.515 | 3,515 | 0,223 | 12 | 63 | 100 | Find by MRM | 18 | 228,1 | 4 |  |  |  |  |
| Cpd 2: dA 6.128 | 6,128 | 0,499 | 174 | 1128 | 100 | Find by MRM | 20 | 252,1 | 2 | dA ESTD | 1094,0 | 100±2,76 pg/uL | - |
| Cpd 2: dA 6.192 | 6,192 | 0,383 | 181 | 1107 | 100 | Find by MRM | 20 | 252,1 | 2 |  |  |  |  |
| Cpd 2: dA 6.183 | 6,183 | 0,423 | 175 | 1083 | 100 | Find by MRM | 20 | 252,1 | 2 |  |  |  |  |
| Cpd 2: dA 6.189 | 6,189 | 0,447 | 163 | 1058 | 100 | Find by MRM | 20 | 252,1 | 2 |  |  |  |  |
| Cpd 1: dA 6.295 | 6,295 | 0,423 | 416 | 2614 | 100 | Find by MRM | 20 | 252,1 | 1 | dA Sample | 2681,5 | - | **245,1±0,04 pg/uL** |
| Cpd 1: dA 6.640 | 6,64 | 0,43 | 436 | 2749 | 100 | Find by MRM | 20 | 252,1 | 1 |  |  |  |  |
| Cpd 3: dG 6.799 | 6,799 | 0,28 | 42 | 364 | 100 | Find by MRM | 20 | 268,1 | 3 | dG ESTD | 370,8 | 100±3,26 pg/uL | - |
| Cpd 3: dG 6.903 | 6,903 | 0,313 | 60 | 376 | 100 | Find by MRM | 20 | 268,1 | 3 |  |  |  |  |
| Cpd 3: dG 6.893 | 6,893 | 0,383 | 60 | 385 | 100 | Find by MRM | 20 | 268,1 | 3 |  |  |  |  |
| Cpd 3: dG 6.878 | 6,878 | 0,435 | 54 | 358 | 100 | Find by MRM | 20 | 268,1 | 3 |  |  |  |  |
| Cpd 2: dG 6.973 | 6,973 | 0,371 | 21 | 146 | 100 | Find by MRM | 20 | 268,1 | 2 | dG Sample | 138,0 | - | **37,22±0,08 pg/uL** |
| Cpd 2: dG 7.252 | 7,252 | 0,276 | 22 | 130 | 100 | Find by MRM | 20 | 268,1 | 2 |  |  |  |  |
