# Supplementary figures and images for: Long-read sequencing reveals absence of 5mC in Ogataea parapolymorpha DL-1 genome and introduces telomere-to-telomere assembly
Source: Front Genet. 2025 May 9;16:1574332. doi: 10.3389/fgene.2025.1574332 (PMC12098377; doi:10.3389/fgene.2025.1574332)

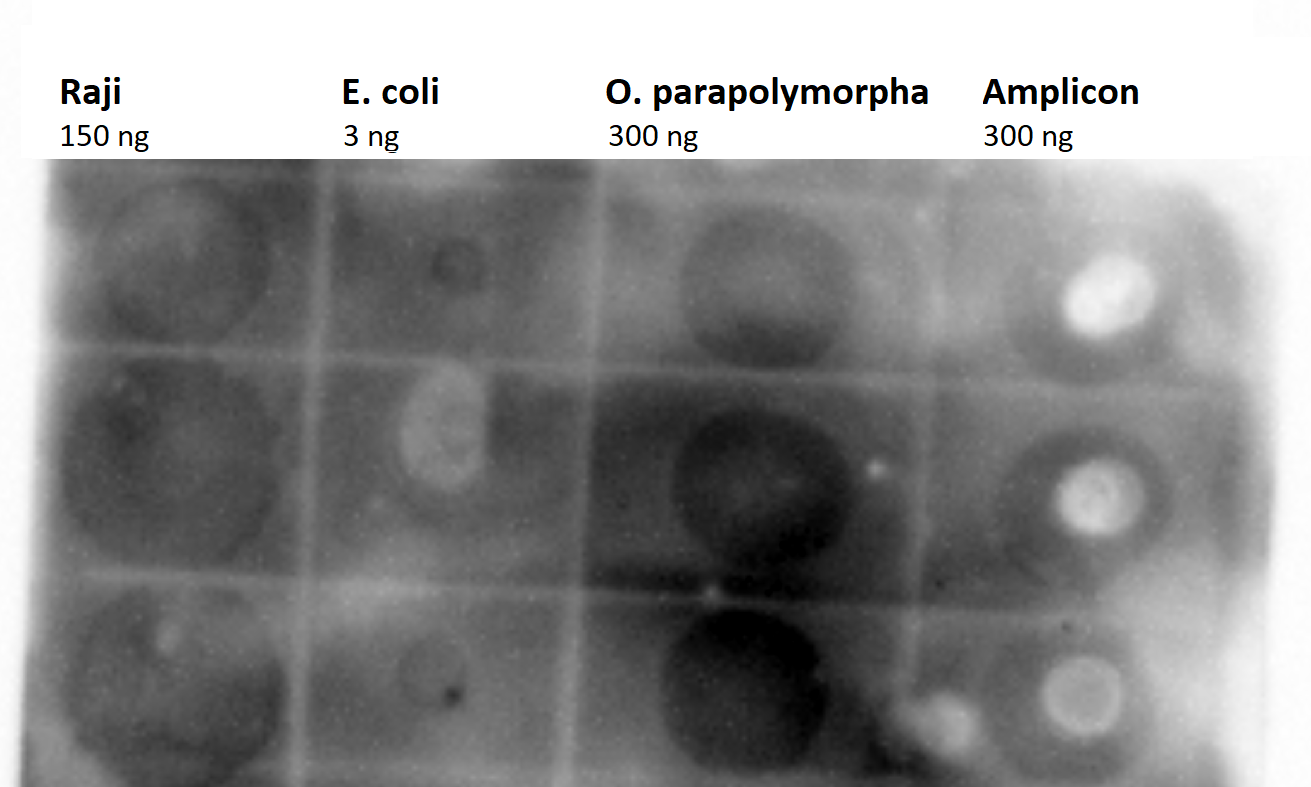

Supplement: Supplementary file 3 [file Image2.png]

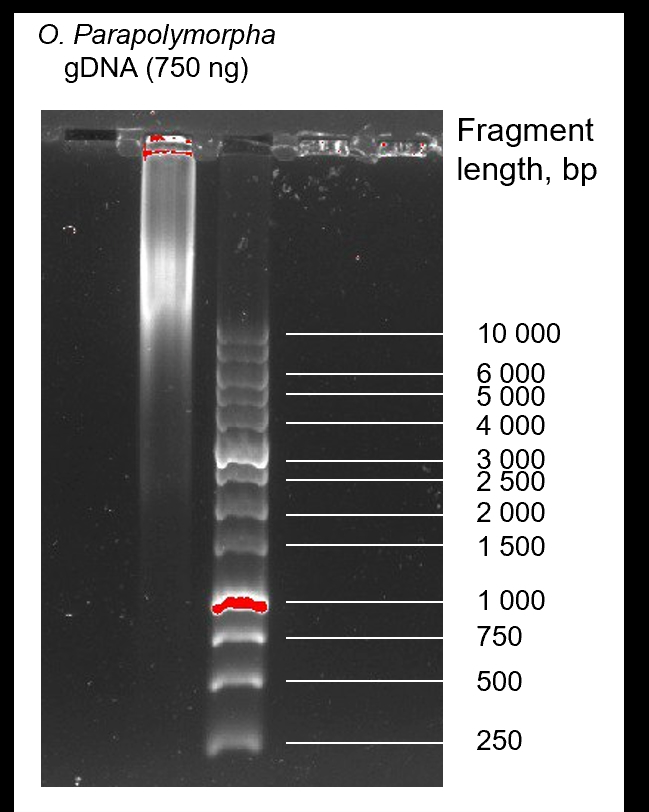

Supplement: Supplementary file 4 [file Image1.jpg]
